# Usefulness of NT-proBNP in dogs with heartworm: could this biomarker be useful to evaluate pulmonary hypertension?

**DOI:** 10.1186/s13071-023-05873-3

**Published:** 2023-08-17

**Authors:** Noelia Costa-Rodríguez, Sara N. García-Rodríguez, Jorge I. Matos, Yaiza Falcón-Cordón, Rodrigo Morchón, José A. Montoya-Alonso, Elena Carretón

**Affiliations:** 1 Internal Medicine, Faculty of Veterinary Medicine, Research Institute of Biomedical and Health Sciences (IUIBS), University of Las Palmas de Gran Canaria, Las Palmas de Gran Canaria, Spain; 2 Zoonotic Diseases and One Health Group, Laboratory of Parasitology, Faculty of Pharmacy, University of Salamanca, 37007 Salamanca, Spain

**Keywords:** Natriuretic peptides, NT-proBNP, Pulmonary hypertension, Biomarker, Heartworm, *Dirofilaria immitis*, Dogs, Animal diseases, Endarteritis

## Abstract

**Background:**

In recent years, the usefulness of echocardiography and serum biomarkers in the diagnosis of pulmonary hypertension (PH) in dogs with heartworm disease has been studied. Previously, N-terminal pro B-type natriuretic peptide (NT-proBNP) has shown high concentrations in dogs with heart disease and/or PH as well as its usefulness as a prognostic indicator, but it has never been evaluated in the diagnosis and prognosis of PH in dogs with heartworm disease. The aim was to evaluate the serum concentrations of NT-proBNP in dogs infected by *Dirofilaria immitis* to determine its usefulness as a tool to detect precapillary PH.

**Methods:**

NT-proBNP was determined in 50 heartworm-infected dogs. Presence/absence of PH was determined echocardiographically, using the Right Pulmonary Artery Distensibility Index (RPAD Index) and the systolic flow of tricuspid regurgitation mainly, together with other echocardiographic measurements following the guidelines of the American College of Veterinary Internal Medicine (ACVIM) for the diagnosis of PH. Other epidemiological parameters (breed, age, sex, status: client-owned or shelter dogs) and clinical parameters (microfilaremia, parasite burden, presence of symptoms, body condition) were collected as well.

**Results:**

Moderate-severe PH was present in 40% of the dogs (RPAD Index < 29.5%), NT-proBNP concentrations being significantly higher compared with dogs that did not have PH. A cutoff for NT-proBNP of ≥ 1178.45 pmol/l showed a sensitivity of 64.3% and a specificity of 95.5% for the presence of moderate-severe PH. Older dogs and dogs from shelters showed significantly higher NT-proBNP concentrations. Dogs with symptoms and low body condition presented significantly higher NT-proBNP concentrations as well.

**Conclusions:**

The determination of NT-pro-BNP concentration can be a useful tool in the diagnostic work-up of dogs with heartworm disease and associated PH and can help to identify animals in the more advanced stage of this disorder.

**Graphical abstract:**

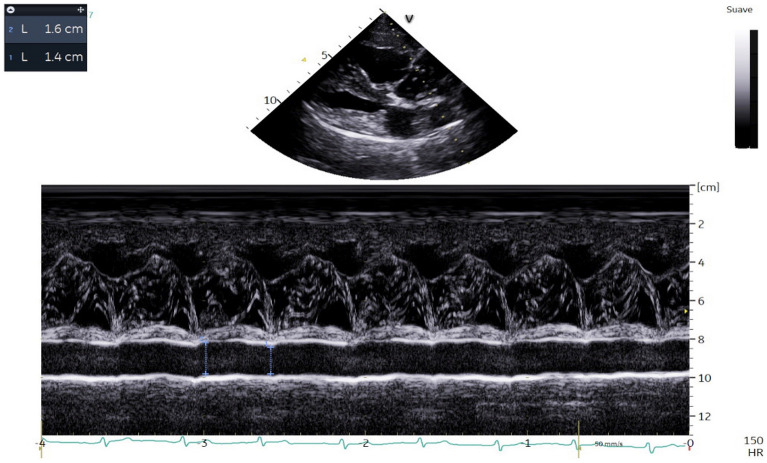

## Background

Heartworm disease, caused by the nematode *Dirofilaria immitis*, is a vector-borne disease and is widely distributed. While dogs and wild canids are definitive hosts, other species, including humans, can also be infected [1]. Infected dogs can remain asymptomatic in less severe infections or may display a mild cough, while in severe infections, they usually present severe clinical signs such as weight loss, dyspnea, ascites, and syncope, being a life-threatening disease [1, 2]. The adult worms are in the pulmonary arteries, causing proliferative endarteritis; these vascular changes start very soon after the arrival of the worms and manifest as vascular inflammation, endothelial damage, sloughing, and villous proliferation of the intima, among others [3, 4]. This situation causes a decrease in the elasticity and increase in the resistance of the arteries that, chronically, can cause the development of pulmonary hypertension (PH) and, in some cases, also right-sided congestive heart failure [1, 2].

PH is a hemodynamic and pathophysiological state present in a wide variety of cardiovascular, respiratory, and systemic diseases [5]. Specifically, heartworm disease generates PH of precapillary origin, which is defined as the result of pathological abnormalities on the arterial side of the pulmonary vascular system. Although the gold standard of PH diagnosis is the right-heart catheterization directly measuring the systolic pulmonary arterial pressure, in veterinary and human medicine, the diagnosis is currently echocardiographycally determined [6].

However, echocardiography is still an indirect method of PH estimation, and there are limitations that make the diagnosis of this condition difficult [7]. Therefore, different non-invasive diagnostic methods for approaching the determination of PH are continually being sought, such as the determination of different serological biomarkers [8, 9]. Among these biomarkers, the concentrations of N-terminal pro-B-type natriuretic peptide (NT-proBNP) have been evaluated in dogs and humans [9, 10]. Brain natriuretic peptide (BNP) exists as a prohormone that is cleaved into the inactive N-terminal fragment and biologically active hormone BNP prior to release into the blood circulation in response to ventricular myocyte stretch [10]. NT-proBNP has a longer lifetime and is typically used as a surrogate marker for the biologically active form [11]. Previous investigations showed that serum concentrations of NT-proBNP act as an excellent biomarker for the diagnosis and monitoring of congestive heart failure and, indirectly, the myocardial function of dogs and cats [12, 13], and elevated NT-proBNP concentrations have been described in dogs with post-capillary PH due to left heart failure secondary to severe mitral regurgitation [14]. Historically, the left ventricle has been considered the major source of BNP, with the right ventricle having a smaller contribution [15]. However, in humans and dogs, increased NT-proBNP concentrations have been described in presence of precapillary PH, which could be useful to stratify the severity of the disease, monitor the response to treatment and serve as an indicator of prognosis [16–18]. Therefore, the aim of this study was to evaluate the serum concentrations of NT-proBNP in dogs infected by *D. immitis* to determine its usefulness as a tool to detect precapillary PH.

## Methods

This prospective study included 50 heartworm-infected dogs brought to the Veterinary Teaching Hospital of the University of Las Palmas de Gran Canaria, located in a hyperendemic region, during the period from September 2021 to July 2022 [19]. Heartworm diagnosis was determined using a commercial ELISA test kit to detect circulating antigens (Uranotest Dirofilaria, Urano Vet SL, Barcelona, Spain) following the manufacturer’s instructions. The dogs were further evaluated for the presence/absence of circulating microfilariae using a modified Knott test.

A complete record was kept for each animal, including demographic and epidemiological data (breed, sex, age, status: client-owned or shelter dogs). All dogs were subjected to history and a complete physical exam. Infected dogs were considered symptomatic if presence of one or more symptoms related to heartworm disease were observed (dyspnea, cough, exercise intolerance, weakness, loss of weight and syncope) as well as symptoms related to right-sided congestive heart failure (ascites, jugular venous distension and hepatomegaly). Their body condition was determined based on the nine-scale body condition score (BCS) system [20], always carried out by the same researcher.

Inclusion criteria were: the dogs had not previously received heartworm chemoprophylaxis, the dogs had not started any treatment against *D. immitis*, and the animal owners provided informed consent to include the dogs in the study. Dogs with concomitant cardiorespiratory diseases (i.e. left heart disease, dilated cardiomyopathy, congenital diseases, chronic respiratory diseases), detected though clinical history, anamnesis, clinical findings and additional diagnostic tests, were excluded from the study. Moreover, to rule out any influence of azotemia, anemia or systemic hypertension in the concentrations of NT-proBNP [7, 21], dogs with abnormal concentrations of serum creatinine and/or blood urea nitrogen (BUN), abnormalities in hematology analysis indicating signs of anemia or systolic blood pressure > 160 mmHg (as measured by oscillometric methods) were also excluded from the study.

All dogs were echocardiographically evaluated, using ultrasound equipment with spectral and color Doppler and multifrequency probes (2.5–10 MHz, Viviq Iq®, General Electric, Boston, MA, USA). All dogs were conscious, gently restrained in right and left lateral recumbency and under electrocardiographic monitoring during the exam. All recordings were made by the same researcher. The presence/absence of PH was determined following the guidelines of the American College of Veterinary Internal Medicine (ACVIM) [5]. The Right Pulmonary Artery Distensibility Index (RPAD Index) and tricuspid regurgitation systolic flow, among other measures taken routinely, were used as previously described [22–24]. Other echocardiographic measurements carried out were tricuspid regurgitation pressure gradient (TRPG), pulmonary trunk to aorta ratio (PT:Ao), global tissue Doppler imaging (G-TDI) index, tricuspid annular plane systolic excursion (TAPSE), pulmonary vein to pulmonary artery ratio (PV:PA), right ventricular acceleration time (AT), right ventricular ejection time (ET), AT:ET ratio, right atrial area index (RAAi) and right ventricular end-diastolic area index (RVEDAi).

For the RPAD index and PV:PA ratio, the dogs were placed in right lateral recumbency; the transducer was placed in the third intercostal space, and the beam was directed caudally and dorsally. The systolic dimension of the pulmonary artery was measured at the maximum diameter and diastolic diameter at its smallest dimension. Diameters of the pulmonary artery were calculated by using the method according to previous authors [25]. For the measurement of tricuspid regurgitation systolic flow, TRPG, PT:Ao ratio, G-TDI, TAPSE, AT, ET, AT:ET ratio, RAAi and RVEDAi, apical four-chamber view and left cranial transverse view were used [26].

For each measurement, three continuous cardiac cycles were recorded. The animals were classified with the presence of moderate-severe PH when presented values of a RPAD Index < 29.5%, a tricuspid regurgitation systolic flow > 3.4 m/s, TRPG < 30 mmHg, PA:Ao ratio > 1.05–1.23, AT > 5.50 ± 0.31, ET > 10.6 ± 0.14, AT:ET ratio < 0.30, G-TDI > 11.8 ± 8.50, RVEDAi > 4.9–10.9 cm^2^/m^2^, RAAi > 4.2–10.2 cm^2^/m^2^ and TAPSE > 4.78–7.64. The parasite load was echocardiographically determined according to previous guidelines [27], scoring the load from 1 to 4, scores 1 and 2 being considered as showing low parasite burden and scores 3 and 4 high parasite burden.

For the determination of NT-proBNP, blood samples were collected from the cephalic vein, placed in serum tubes and centrifuged at 1432 g for 10 min. The samples were kept refrigerated until analysis, always within the next 2 h. NT-proBNP was measured with the VCHECK immunochromatography analyzer (Bionote, Big Lake, MN, USA), previously validated for canine species [28]. Reference values for healthy dogs were established by the manufacturer as < 900 pmol/l, based on values suggestive of cardiac disease [28].

Statistical analyses were performed using commercially available software (BM SPSS® Statistics 25.0, New York, USA). For categorical variables (breed, sex, legal status, BCS, symptoms, presence/absence of microfilariae, parasite load, presence/absence of PH), frequencies and percentages were analyzed; for continuous variables (age, NT-proBNP concentrations), standard deviation, median and interquartile range are shown. For continuous variables, the differences in the parameters between groups were evaluated by means of Mann-Whitney/Kruskall-Wallis tests (non-parametric) or t-Student/ANOVA (parametric) based on the normality of the variables to be evaluated by means of Shapiro-Wilks test. For categorical variables, the differences were evaluated with Pearson's non-parametric chi-square test. For multiple comparisons, when significant differences were identified, post hoc pairwise comparisons were made using Pearson's *P* test with Bonferroni corrections. All contrasts were accompanied by the effect size estimator to complete the interpretation of the results. For categorical variables, Cramer's V was used and for continuous variables, Cohen's D. Receiver-operating characteristic (ROC) curve analyses were performed to determine the optimal cutoff values for the prediction of RPAD index < 29.5% (moderate or severe PH). For all analyses, *P* < 0.05 was considered statistically significant. The results of the ROC curves of the NT-proBNP concentrations were used to estimate the event of suffering from PH (RPAD index < 29.5%), and the cut-off points of the parameters that maximize sensitivity and specificity (through of the Youden index) were evaluated.

For this study, no ethical approval was required, since all blood samples were routinely collected for official diagnostic and monitoring purposes and subsequently made available for this study. The study was carried out in accordance with the current Spanish and European legislation on animal protection.

## Results

Most of the studied dogs were mongrels (58%, *n* = 29) while 42% (*n* = 21) were pure-bred dogs. The most representative pure-bred dogs were Canarian hound, German shepherd, Siberian husky and Chihuahua. The proportion of females was higher than that of males (54% versus 46%, respectively). The mean age of the studied dogs was 4.6 years, ranging from 1 to 14 years. Based on their legal status, 72% (*n* = 36) were client-owned dogs, and 28% (*n* = 14) were from animal shelters. Regarding BCS, dogs were classified as very thin/underweight (BCS 1 to 3) (24%, *n* = 12), normoweight (BCS 4–5) (64%, *n* = 32) or overweight/obese (BCS 6 to 9) (12%, *n* = 6) [17].

Symptoms were present in 38% (*n* = 19) of the dogs. When microfilaremia was assessed, 48% (*n* = 24) were microfilaremic and 52% (*n* = 26) amicrofilaremic. Regarding parasite load, this was established as high in 50% (*n* = 25) of the dogs and low in 50% (*n* = 25).

Dogs were classified into two groups according to presence or absence of PH. Moderate-severe PH was present in 40% (*n* = 20) of the dogs and mild or absent in 60% (*n* = 30). Mean RPAD index in dogs with PH was 20.1%, and mean RPAD index in dogs without PH was 40.6%. All dogs with tricuspid regurgitation systolic flow > 3.4 m/s already showed RPAD indexes < 29.5%. No significant differences were observed by breed or sex regarding PH status although the results showed that dogs with moderate-severe PH were significantly older (Mann-Whitney test, *U* = 456.50, *Z* = − 3.82, *P* = 0.002). Based on their legal status, significant differences were observed between both groups when compared with the PH status (ANOVA, *F* = 14.84, *P* = 0.034). Neither microfilaremia (Chi-square test, *X*^2^ = 1.09, *df* = 1, *P* = 0.297) nor parasite load (Chi-square test, *X*^2^ = 1.33, *df* = 1, *P* = 0.248) was statistically influenced by the presence/absence of PH (*P* > 0.05).

No significant differences were observed by breed or sex regarding NT-proBNP concentrations. Dogs with pathological concentrations of the biomarker (> 900 pmol/l) were significantly older [8.1 (7.2–8.9) years] compared with dogs with NT-proBNP concentrations within reference values [6.5 (5.7–7.4) years] (ANOVA, *F* = 13.52, *P* = 0.002). Neither microfilaremia nor parasite load was statistically influenced by the results of NT-proBNP concentrations. NT-proBNP was significantly higher in dogs with PH (2004.5 pmol/l, 1249.5–2759.4 pmol/l) compared to dogs without PH (689.9 pmol/l, 528.5–851.2 pmol/l) (ANOVA, *F* = 13.28, *P* = 0.001). Moreover, the concentrations of NT-proBNP in dogs with PH were significantly above the reference values, while NT-proBNP did not differ significantly from the reference values in dogs with mild or absence of PH.

NT-proBNP concentrations were significantly higher in dogs with symptoms (1749.8 pmol/l, 1176.5–2323.1 pmol/l), being more than three times higher than in dogs without symptoms (573.1 pmol/l, 233.8—912.4 pmol/l) (ANOVA, *F* = 13.50, *P* = 0.001). Significant differences were also found in NT-proBNP concentrations in dogs with lower BCS (1478.8 pmol/l, 1105.2–1852.3 pmol/l) compared to dogs with normoweight (923.5 pmol/l, 631.4–1215.6 pmol/l) or overweight/obesity (819.2 pmol/l, 602.26–1036.24 pmol/l) (ANOVA, *F* = 5.83, *P* = 0.02).

Using as cutoff the reference value of NT-proBNP for healthy dogs provided by the manufacturer (900 pmol/l), the sensitivity of NT-proBNP to detect PH was 64.3%, with a specificity of 86.4%. For the determination of presence of moderate-severe PH, a cutoff of ≥ 1178.45 pmol/l showed a sensitivity of 64.3% and a specificity of 95.5% for the presence of moderate-severe PH. The area under the ROC curve (AUC) of the NT-proBNP concentrations to estimate the presence of PH was moderate (0.747, 95% IC: 0.543, 0.950, Youden's J statistic 0.597).

## Discussion

Many of the biomarkers evaluated in dogs with cardiac disease have also been evaluated as tools in diagnosing PH, including NT-proBNP. Many studies have explored NT-proBNP concentrations in dogs with left ventricular dysfunction and/or postcapillary PH [16, 29], although there are only a few studies in veterinary medicine briefly describing an elevation in NT-proBNP concentration in dogs with precapillary PH [7, 16]. However, it is well studied in humans that NT-proBNP concentrations are significantly elevated in the presence of precapillary PH, which causes right ventricular stress and pressure overload with the release of BNP from the right ventricular myocardium [10, 17, 18]. Moreover, in humans, increases in NT-proBNP concentrations have been correlated to presence and severity of precapillary PH, and NT-proBNP concentration serves as a diagnostic support, as a tool to monitor the response to treatment and as a prognostic predictor of mortality [17, 18, 30].

The results of the present study demonstrate the utility of this biomarker in helping to determine the presence of PH in dogs infected with *D. immitis*, being consistent with the results reported by other authors in dogs with precapillary PH caused by other pathologies of various origins [7]. Although PH is a serious and frequent phenomenon in heartworm infection, and this may be irreversible even once the parasites are eliminated [31], the usefulness of this biomarker for detection and assessment of PH had never been evaluated for this disease to our knowledge. There is only one published study in which NT-proBNP was evaluated in dogs with heartworm, pathological concentrations in the chronic stages of the disease (classes III and IV) being described, being useful to help establish severity status [32]. Although PH was not evaluated in that study, which could be the key piece that explains the increases in NT-proBNP, the authors argued that presence of PH, cardiac damage and pulmonary thromboembolisms may be the cause of the increases of this biomarker. Likewise, an older study established a relationship between elevations of another natriuretic peptide, atrial natriuretic peptide (ANP), and the presence of mild heartworm infection in dogs [33]. Moreover, other biomarkers have shown their usefulness in the determination of PH in dogs with *D. immitis*, such as, endothelin-1, C-reactive protein and other acute phase proteins [34–37].

The cutoff points that maximize the sensitivity and specificity for the detection of PH were established at 1178.75 pmol/l (64.3% and 95.5%, respectively), the sensitivity and specificity being lower for the cutoff of 900 pmol/l established by the laboratory (64.3% and 86.4%, respectively). These results differ from those shown by other authors, in which the sensitivity and specificity of NTproBNP concentration was 91.7% and 62.5%, respectively, for an NT-proBNP concentration cutoff of 900 pmol/l [7]. This may be due to the different methodology used, highlighting the use of a different NT-proBNP analyzer and the estimation of the presence of PH using other echocardiographic indicators. Given that the reference values ​​provided by laboratories are based on the diagnosis of cardiac damage, the value of 1178.75 pmol/l could be interesting in helping to determine the presence/absence of PH, especially because of the high specificity achieved in this research for that cutoff.

The results showed that older dogs had a higher incidence of PH and therefore significantly higher values ​​of NT-proBNP, probably due to older dogs having more chronic infections and therefore more severe and advanced vascular damage. Similarly, dogs from shelters presented higher NT-proBNP values, possibly because they mostly had had a poorer quality of life and usually came from rural areas, where they are often used as working animals (i.e. hunting or guard dogs) and housed outdoors without prevention against mosquito vector bites [38].

However, no significant changes in natriuretic peptide concentrations and presence/absence of PH were observed between microfilaremic and non-microfilaremic dogs. These results were similar to previous studies in which no relationship was observed between the presence of microfilariae and PH [23]. These results apply equally to the parasite load, there being no direct relationship with the presence of PH or elevations of NT-proBNP. Although there are still discrepancies in this regard, these results coincide with previous studies that indicate that the parasite load is not a determining factor in the development of a severe proliferative endarteritis [23, 27, 39].

Symptomatic patients showed NT-proBNP values ​​three times higher than those observed in asymptomatic patients. This could be because symptomatic dogs usually present more advanced and chronic stages of the disease; therefore, a greater presence of PH severity was to be expected. These results also apply to dogs with low BCS, which also showed significantly higher NT-proBNP values, probably due to the characteristic weight loss suffered by dogs with HW in more advanced stages. However, another study did not find differences in the presence or absence of PH according to body score, so it is necessary to do more studies in this regard [37].

## Conclusions

The determination of NT-pro-BNP concentration can be a useful tool in the diagnostic work-up of dogs with heartworm disease and associated PH and can help to identify animals in the more advanced stage of this disorder. It is necessary to evaluate the cardiopulmonary status of the dog as a determining factor to choose a specific treatment protocol and provide an accurate prognosis. The determination of NT-proBNP could be very helpful in this regard. Further studies with a large number of animals are necessary to determine optimal cut-off values ​​for diagnosis with greater strength as well as to evaluate the utility at the prognostic level, as has already been established in humans.

## Data Availability

All data generated or analysed during this study are included in this article. The datasets used and/or analysed during the present study are available from the corresponding author upon reasonable request.

## Abbreviations

PH: Pulmonary hypertension
NT-proBNP: N-terminal pro B-type natriuretic peptide
